# Salvage radiotherapy strategy and its prognostic significance for patients with locoregional recurrent cervical cancer after radical hysterectomy: a multicenter retrospective 10-year analysis

**DOI:** 10.1186/s12885-023-11406-z

**Published:** 2023-09-26

**Authors:** Minjie Shan, Yuping Deng, Wen Zou, Shasha Fan, Yanlong Li, Xianling Liu, Jingjing Wang

**Affiliations:** 1 Department of Oncology, The Second Xiangya Hospital of Central South University, 139 Middle Renmin Road, Changsha, 410011 Hunan People’s Republic of China; 2 Department of Gynecologic Oncology, The Affiliated Cancer Hospital of Xiangya School of Medicine, Hunan Cancer Hospital, Central South University, Ward 5, Hunan, People’s Republic of China; 3 Oncology Department, The First Affiliated Hospital of Hunan Normal University, Hunan Provincial People’s Hospital, Hunan, People’s Republic of China; 4 Oncology Department, Shanxi Provincial People’s Hospital, Shanxi, People’s Republic of China

**Keywords:** Cervical cancer, Radiotherapy, Systemic inflammation response index, Prognosis, Recurrence

## Abstract

**Objective:**

We aimed to evaluate the clinical efficacy and prognostic significance of intensity-modulated radiotherapy (IMRT)-based salvage concurrent chemoradiotherapy (CCRT) for patients with locoregional recurrence cervical cancer after radical hysterectomy and evaluated two salvage radiotherapy modes—regional RT (involved-field RT combined with regional lymph nodes) and local RT (involved-field RT).

**Methods:**

Patients were enrolled retrospectively from January 2011 to January 2022 in three medical centers. Clinical outcomes were analyzed using the Kaplan–Meier method and a Cox proportional hazards model. Propensity score (PS) matching analysis was used to compare the two RT groups.

**Results:**

There were 72 patients underwent IMRT-based salvage CCRT. The 5-year overall survival and progression-free survival rates were 65.9% and 57.6%, respectively. Univariate analysis showed that patients with stump recurrence, a lower systemic inflammation response index (SIRI), only one metastatic lesion, and received regional RT had better prognosis than their counterparts. In multivariate analysis, recurrence site was the independent prognostic factor of OS, and SIRI was that of PFS. After PS matching, there were 15 patients each in the regional RT group and local RT group. The 5-year OS rate of regional RT group was better than that of local RT group (90.9 vs. 42.4, p = 0.021). However, there was no significant difference between them in terms of PFS rate (47.1 vs. 38.1, p = 0.195).

**Conclusion:**

Locoregional recurrent cervical cancer treated with IMRT-based salvage therapy has a good prognosis. Recurrence site and SIRI were independent prognostic factors. Regional RT may be a better option for patients with locoregional recurrent.

**Supplementary Information:**

The online version contains supplementary material available at 10.1186/s12885-023-11406-z.

## Background

Cervical cancer is the most common gynecologic cancer in China and has a higher incidence than ovarian and endometrial cancers [1]. Radical surgery is the standard treatment for early-stage cervical cancer [2]. Most early-stage patients have good prognosis; however, there are approximately 10% of these patients experience recurrence [3, 4]. The recurrence patterns vary depending on specific clinicopathological characteristics of cervical cancer, such as postoperative risk factors, adjuvant therapy, and methods of surgery [5, 6]. Patients with postoperative endometrial infiltration are prone to distant metastasis, while patients with positive parametrial margin are prone to pelvic and supraclavicular/paratracheal lymph node metastasis, and patients with positive lymph node are prone to pelvic and abdominal lymph node metastasis [3]. However, the most common recurrence site is still the pelvic cavity, followed by the retroperitoneal and distant lymph nodes and other organs [3, 7, 8]. A study showed that in recurrent cervical cancer patients, local recurrence accounts for 46.8%, distant metastasis accounts for 34%, and lymph node metastasis accounts for 51.1%. The most common site of metastasis is pelvic, accounting for about 76%, followed by paraaortic lymph nodes, accounting for 29.8% [9].

For patients with locoregional recurrence after radical surgery who have not received previous radiotherapy (RT), salvage concurrent chemoradiotherapy (CCRT) is recommended by National Comprehensive Cancer Network (NCCN) [2, 10]. Studies have shown that the 3-year OS of patients with locoregional recurrence cervical cancer and receiving CCRT is 58–85% [11, 12].

Radiotherapy for cervical cancer has a long history. In 1904, American surgeon Robert Abbe first used radium alone to treat cervical cancer []. In the 1910s, brachytherapy began to sprout . During the decade from 1950 to 1960, randomized trials established many principles for cervical cancer radiotherapy, and the treatment model of combining external irradiation and brachytherapy gradually matured []. Until 1999, based on the results of five clinical studies [14–18], CCRT were recommended as the standard treatment for locally advanced cervical cancer by the National Cancer Institute (NCI). However, in that era, the conventional RT, such as 4-field techniques and simple opposing (anterior-posterior) techniques, were generally used for external-beam radiation. With the development of radiotherapy technology, it has led to an era of intensity modulated RT (IMRT),, which is superior to conventional RT in terms of toxicities, even prognosis [19]. In both of postoperative adjuvant and definitive radiotherapy for cervical cancer, no statistically significant difference was observed between the IMRT group and the conventional RT group in terms of prognosis, however IMRT group was significantly lower than conventional RT group in terms of gastrointestinal and urinary toxicity [20–22]. In patients with local pelvic recurrence of cervical cancer after radical surgery, a retrospective study showed that the IMRT patients experienced less acute and chronic gastrointestinal and urinary toxicity and better short-term effects and 5-year OS and PFS compared to those treated with conventional RT [12]. However, this study did not include patients with retroperitoneal recurrence. Thus, the prognosis of patients with locoregional recurrence treated with IMRT-based salvage therapy should be explored again.

Moreover, the RT mode to be selected for CCRT in patients with locoregional recurrence of cervical cancer remains undetermined. At present, there are two common RT modes: involved-field RT combined with regional lymph nodes (regional RT) and involved-field RT alone (local RT) [11, 23, 24]. Many studies have shown that RT covering both the regional lymph node area and gross tumor is an appropriate RT mode [25, 26]. However, this mode of RT confers considerable side effects in patients, such as bone marrow suppression, genitourinary, and gastrointestinal toxicity. Another RT mode involving radiation to the gross tumor only has been shown to lead to a good prognosis (4-year overall survival : 50.1%; 4-year local control (LC): 67.4%) and result in few side effects, especially for patients with recurrent lesions measuring less than 17 mm; it is also considered a good alternative for patients with local recurrence [27]. However, the two RT modes are still controversial. Thus, the optimal RT mode for cervical cancer patients with local recurrence needs to be investigated.

Finally, ongoing research is focused on the identification of new prognostic factors. There is increasing evidence that inflammation plays an important role in the development of various tumors [28]. The platelet-to-lymphocyte ratio (PLR) and systemic inflammation response index (SIRI), based on peripheral blood cell counts, are inflammatory response biomarkers. Many studies have associated these biomarkers with the prognosis of several malignant tumors, including operable cervical cancer [29–31]. However, the effect of SIRI and PLR on prognosis of patients with locally recurrent cervical cancer remains unknown.

This study was conducted with an aim to evaluate the survival prognosis of patients treated with IMRT-based salvage therapy. Additionally, there are few studies on the differences in efficacy and adverse effects between regional RT and local RT in the treatment of locoregional recurrence cervical cancer patients. Therefore, this study explores the prognostic differences and novel potential prognostic factor of these two RT modes on patients with locoregional recurrence.

## Methods

### Patients

A cohort of patients diagnosed with cervical cancer were retrospectively examined at the Second Xiangya Hospital of Central South University, Hunan Cancer Hospital, and Hunan Provincial People’s Hospital from January 2011 to January 2022. The following patients were included in the study: (1) those with a pathological diagnosis of cervical cancer, including squamous cell carcinoma and adenocarcinoma, (2) those with locoregional recurrence (≤ 3 lesions) who were initially treated with radical surgery without RT for early-stage cervical cancer, including carcinoma in situ, stage I to stage IIa.

, (3) those with recurrence time of more than 3 years and diagnosis made via biopsy, and (4) those whose follow-up data were complete. The following patients were excluded from the study: (1) those with other tumors, infectious diseases, hematological diseases, or severe liver or renal dysfunction; (2) those who received adjuvant RT; (3) those who had distant metastasis; or (4) those for whom local treatment was not available. The last follow-up was conducted on January 1, 2023. Recurrence was confirmed based on clinical, imaging, and pathological evidence. The locoregional recurrence mentioned here refers to vaginal stump, pelvic, or retroperitoneal lymph node recurrence. During routine follow-up, patients with new lymph node involvement measuring more than 1 cm detected on computed tomography (CT) or based on abnormal fluorodeoxyglucose uptake on positron emission tomography (PET) were considered to have recurrence. The newly punctured pathological specimen was verified with the previous pathology and determined as recurrent cervical cancer. The disease stage was defined according to the International Federation of Gynecology and Obstetrics (FIGO) 2009 staging system. All methods were performed in accordance with the relevant guidelines and regulations, and informed consent was obtained from all subjects and/or their legal guardian(s).

### Radiotherapy and chemotherapy

Regional RT refers to irradiation of the gross tumor and prophylactic irradiation of the lymphatic drainage area. Local RT refers to irradiation of the gross tumor alone. In general, the RT mode was selected according to the performance status and timing of recurrence. Vaginal stump recurrence generally necessitated regional RT including brachytherapy. Elderly patients or patients with postoperative complications and patients with a prolonged recurrence time (more than 2 years) generally received local treatment. Otherwise, regional RT was administered. IMRT was used for external irradiation, which was planned with the Varian Eclipse Treatment Planning System 11.0 (Varian Medical Systems, Palo Alto, CA, USA) and delivered with 6-MV X-rays using a Varian 23EX. For regional RT, the gross tumor volume (GTV) included all detectable involved areas of recurrent disease confirmed using CT or PET, and the clinical target volume (CTV) included the lymphatic drainage area. For local RT, only the GTV was considered. The planning target volume was delineated by margins of 7–10 mm around the GTV and CTV. Cone-beam CT was performed once a week. For CTV of the pelvic lymph node field, the superior margin was set at the L4–L5 intervertebral space, and the inferior margin was set at the lower edge of the obturator. For CTV of the para-aortic lymph node field, the superior margin was set at the renal vascular level, and the inferior margin was located between L5 and S1. The CTV dose was 45–50 Gy, with 1.8 or 2 Gy administered daily, and the GTV dose was 60–66 Gy. When the vaginal stump was the site of recurrence, brachytherapy (192Ir) was used to boost external-beam RT doses. According to the depth and size of tumor invasion, a supplement of 18–30 Gy was administered [32].

Concurrent chemotherapy was provided. Patients with good physical status received double agent regimen of cisplatin 50–70 mg/m^2^ plus paclitaxel 135–175 mg/m^2^ every three weeks for maximum 6 cycles, and patients with bad physical status received single cisplatin 50–70 mg/m^2^ every three weeks for maximum 4 cycles.

### Data collection

Clinical information, including age, initial nodal size, operation style, initial stage and treatment, pathological pattern, time to recurrence, recurrence sites, number of recurrence lesions, RT mode, and results of routine blood tests at the time of recurrence, was collected. PLR and SIRI were calculated using the following formulae: PLR = platelet count/lymphocyte count and SIRI = neutrophil count × monocyte count/lymphocyte count [29, 33].

### Statistical analysis

The SPSS software (version 26.0; SPSS, Inc., Chicago, IL, USA) was used for all statistical analyses. The major endpoint was OS calculated from the date of diagnosis until the date of death or final follow-up. Progression‐free survival (PFS) was defined as survival from the date of diagnosis until the date of (1) disease progression, (2) relapse, (3) mortality from any cause, or (4) final follow‐up.

The cutoff values for PLR and SIRI were calculated using receiver operating characteristic (ROC) curves, and the optimal cutoff value was defined according to the maximum Youden index [34]. Fisher’s exact test and χ2 test was used to determine whether there was a correlation between two variables. Univariate analysis was performed using the Kaplan–Meier method to assess the 5-year OS and PFS rates. Statistical differences between survival curves were evaluated using the log-rank test. Multivariate Cox proportional hazards models were created using the input selection technique of factors significant in the univariate analysis. Patients were classified into the regional RT group and local RT group. Treatment outcomes were compared between the two groups after 1:1 ratio propensity score (PS) matching, which was using calipers of width equal to 0.02 of the SD of the logit of the PS, adjusted for age, recurrence sites, recurrence time, number of metastatic lesions, and SIRI. In all tests, *P*-values < 0.05 indicated a significant difference. Hazard ratios (HRs) and their 95% confidence intervals (CIs) were estimated to assess the magnitude of risk. And I confirm that all methods were performed in accordance with the relevant guidelines and regulations.

## Results

### Patient and treatment characteristics

A total of 72 patients were included in the analysis. The median follow-up time was 56.5 months (range, 12–126 months), and the patient characteristics are summarized in Table 1. The median age of the included patients was 51 (range, 34–69) years. According to previous studies, the tumor size of 4 cm was the cutoff value for prognosis and staging [35]. Therefore, we classified the patients into two groups based on the initial tumor size: 48 patients with less than 4 cm tumor and 24 patients with greater than or equal to 4 cm tumor. The median time to recurrence was 21 (4–114) months. In many studies, 24 months was used as the cutoff time to recurrence [36, 37]. The time to recurrence was ≤ 24 months for 41 patients and > 24 months for 31 patients. There were 25 cases of stump recurrence and 47 cases of pelvic and abdominal lymph node recurrence. In the salvage treatment cohort, 56 patients received regional RT, and 16 patients received local RT. All patients were treated with CCRT. A total of 65 patients with good physical status received double agent regimen of cisplatin and paclitaxel, and 7 patients with bad physical status received single cisplatin.

**Table 1:** Clinical characteristics of patients

Variable	Number (%)	Variable	Number (%)
Total	72 (100)	**Recurrence Site**	
**Median Age**	51 Years old	Vaginal stump	25 (34.7)
≤ 51	39 (54.2)	Pelvic and abdominal cavity	47 (65.3)
> 51	33 (45.8)	**No. of Recurrence sites**	
**Initial Operation Style**		1	36 (50.0)
Hysterectomy	12 (16.7)	2 ~ 3	36 (50.0)
Radical Hysterectomy and Pelvic Lymphadenectomy	60 (83.3)	**SIRI**	Cut-off Value:1.185
**Initial Stage**		< 1.185	45(51.1)
Carcinoma in situ	5 (6.9)	> 1.185	43(48.9)
IA	17 (23.6)	**PLR**	Cut-off Value: 142.4
IB	31 (43.1)	< 142.4	28(31.8)
IIA	19 (26.4)	> 142.4	60(68.2)
**Initial tumor size**		**Median Recurrence Time**	21 Months
< 4 cm	48 (66.7)	**Recurrence Time**	
≥ 4 cm	24 (33.3)	≤ 24 months	41 (56.9)
**Pathological Types**		> 24 months, < 60 months	17 (23.6)
Squamous carcinoma	66 (91.7)	≥ 60 months	14 (19.4)
Adenocarcinoma	6 (8.3)	**Radiotherapy Mode**	
**Recurrence Site**		Regional RT	56 (77.8)
Vaginal stump	25 (34.7)	Local RT	16 (22.2)
Pelvic and abdominal cavity	47 (65.3)	**Initial adjuvant chemotherapy**	
**lymph vascular invasion**		Yes	32 (44.4)
Yes	4(5.6)	No	40 (55.6)
No	68(94.4)		

PLR: The platelet-to-lymphocyte ratio

SIRI: Systemic inflammation response index

### Cutoff values for PLR and SIRI

For PFS, the area under the ROC curve (AUC) of PLR was 0.552, whereas that of SIRI was 0.704. The optimal cutoff values were 142.4 and 1.185 for PLR and SIRI, respectively. For OS, the AUC of PLR was 0.470, whereas that of SIRI was 0.628. There was no statistical significance for prognosis. (Supplement file 1).

### Prognostic analysis

**Fig. 1:**
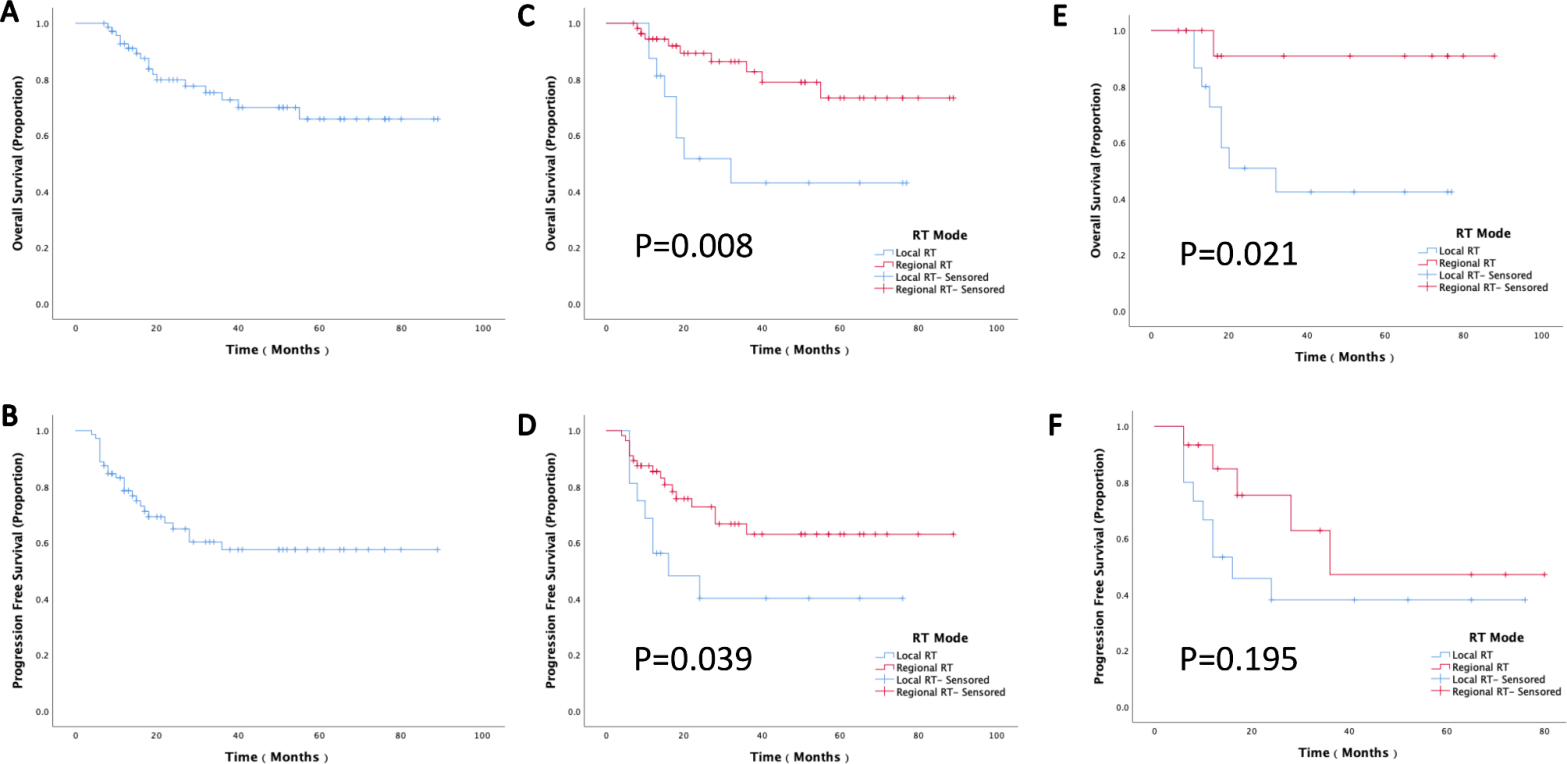
**(a)** Overall survival and **(b)** progression-free survival of patients with locoregional recurrence cervical cancer who received intensity-modulated radiation therapy-based salvage treatment. **(c)** Overall survival and **(d)** progression-free survival of patients receiving regional RT and local RT before matching. **(e)** Overall survival and **(f)** progression-free survival of patients receiving regional RT and local RT after matching

Seventy-two patients with locoregional recurrence received the IMRT-based salvage treatment, and the 5-year OS and PFS rates were 65.9% and 57.6%, respectively (Fig. 1a, b). In the univariate analysis, the recurrence site, RT mode, numbers of metastatic lesions, and SIRI significantly influenced the OS and PFS rates (Table 2). The 5-year OS and PFS rates of patients with stump recurrence were 90% and 87.2%, respectively, which were better than those of patients with pelvic and abdominal recurrence, whose 5-year OS and PFS rates were 52% and 39.5%, respectively. Patients who received regional RT had better prognosis than those who received local RT (OS: 73.3% vs. 43.1%, p = 0.0008; PFS: 63% vs. 40.2%, p = 0.039). The clinical characteristics of the above two groups were compared. It was found that recurrence sites and SIRI between the two groups were not well-balanced (Table 3). After PS matching, there were 15 patients each in the regional RT group and local RT group. All background factors between the two groups were well-balanced (Table 3). The 5-year OS rate of regional RT group was better than that of local RT group (90.9 vs. 42.4, p = 0.021). However, there was no significant difference between them in terms of PFS rate (47.1 vs. 38.1, p = 0.195) (Fig. 1C-F).

**Table 2:** Univariate analysis of overall survival and progression-free survival rates for all patients

Variable	Univariate Analysis			
	5-year OS rate (%)	P-value	5-year PFS rate (%)	P-value
Total	65.9		57.6	
**Age, years**				
≤ 51	66.0		57.2	
> 51	68.6	0.996	60.5	0.826
**Operation Style**				
Hysterectomy	60.6		42.2	
Radical Hysterectomy and Pelvic Lymphadenectomy	67.4	0.396	61.2	0.239
**Initial tumor size**				
< 4 cm	71.1		64.8	
≥ 4 cm	57.8	0.527	43.5	0.174
**Initial Stage**				
Carcinoma in situ	80.0		60	
I	70.5		60.1	
II	55.2	0.791	50.9	0.791
**Pathological Pattern**				
Squamous carcinoma	64.2		56.6	
Adenocarcinoma	83.3	0.529	66.7	0.776
**lymph vascular invasion**				
Yes	66.8		60.2	
No	50	0.801	0.0	0.545
**Recurrence Site**				
Vaginal stump	90.0		87.2	
Pelvic and abdominal cavity	52.0	0.002	39.5	0.002
**No. of Recurrences**				
1	77.4		77.2	
> 1	54.9	0.023	35.8	0.004
**SIRI**				
< 1.185	83.4		75.3	
> 1.185	37.2	0.001	30.4	0.000
**PLR**				
< 142.4	70.1		73.4	
> 142.4	61.7	0.928	48.5	0.111
**Recurrence Time**				
≤ 24 months	65.1		50.5	
> 24 months	65.5	0.959	61.8	0.858
**Radiotherapy Mode**				
Regional RT	73.3		63.0	
Local RT	43.1	0.008	40.2	0.039

**Table 3:** Correlation analysis between radiotherapy modes and clinical features before and after matching

Variable	Before Matching			After Matching		
	Regional RT (56)	Local RT (16)	P Value	Regional RT (15)	Local RT (15)	P Value
**Age**						
≤ 51	30	9		9	8	
> 51	26	7	0.85	6	7	0.713
**Operation Style**						
Hysterectomy	9	3		2	3	
Radical Hysterectomy and Pelvic Lymphadenectomy	47	13	0.80	13	12	0.624
**Initial tumor size**						
< 4 cm	35	13		7	12	
≥ 4 cm	21	3	0.161	8	7	0.128
**Recurrence sites**						
Vaginal stump	23	2		3	2	
Pelvic and abdominal cavity	33	14	0.034	12	13	0.624
**No. of Recurrences**						
1	30	6		6	5	
> 1	26	10	0.257	9	10	0.705
**Recurrence time**						
≤ 24 months	22	9		8	8	
> 24 months	34	7	0.227	7	7	1.0
**Initial Stage**						
Carcinoma in situ	4	1		0	1	
I	38	10		11	9	
II	14	5	0.882	4	5	0.519
**Pathological Types**						
Squamous carcinoma	51	15		13	14	
Adenocarcinoma	5	1	0.732	2	1	0.543
**SIRI**						
< Cut-off Value	35	5		4	5	
> Cut-off Value	21	11	0.027	11	10	0.690

The analysis of SIRI as a prognostic factor showed that a higher SIRI was associated with a poorer prognosis (OS: p = 0.001; PFS: p < 0.001). The correlation between SIRI and clinical characteristics of patients was analyzed (Table 4). SIRI was related to the recurrence site (p = 0.000), number of recurrence lesions (0.004), and RT mode (p = 0.027), which further confirmed that SIRI was related to prognosis.

**Table 4:** Correlation analysis between systemic inflammation response index and clinical features

Variable	SIRI		
	≤ Cut-off Value	> Cut-off Value	P Value
**Age**			
< 51	22	17	
>=51	18	15	0.874
**Operation Style**			
Hysterectomy	7	5	
Radical Hysterectomy and Pelvic Lymphadenectomy	33	27	0.832
**Initial tumor size**			
< 4 cm	30	18	
≥ 4 cm	10	14	0.094
**Recurrence sites**			
Vaginal stump	22	3	
Pelvic and abdominal cavity	18	29	0.000
**Radiotherapy Mode**			
Regional RT	35	21	
Local RT	5	11	0.027
**No. of Recurrences**			
1	26	10	
2–3	14	22	0.004
**Recurrence time**			
≤ 24 months	16	15	
> 24 months	24	17	0.558
**Initial Stage**			
Carcinoma in situ	3	2	
I	27	21	
II	10	9	0.944
**Pathological Types**			
Squamous carcinoma	36	30	
Adenocarcinoma	4	2	0.567

Recurrence site, RT mode, number of recurrence lesions, and SIRI were included in the multivariate regression analysis. Only SIRI influenced the PFS (P = 0.029, HR: 0.347, 95% CI: 0.134–0.898), and recurrence site influenced the OS (P = 0.042, HR: 0.098, 95% CI: 0.011–0.922) (Supplement file 2). SIRI and recurrence site were independent prognostic factors of prognosis in patients with locoregional recurrent cervical cancer. Other factors, such as age, initial stage, tumor size, operation style, and pathological pattern did not influence prognosis.

### Toxicity and patterns of failure

The main toxicities were hematologic toxicity, enteritis, cystitis, fatigue, nausea, and vomiting, and most of the toxicity were of grade 1 or 2. Grade 3 toxicities, including: neutropenia (20.8%), anemia (20.8%), Thrombocytopenia(15.3%), enteritis (18.1%), nausea and vomiting (8.3%), fatigue (6.9%). Patients with high dose (≥ 60 Gy) were more likely to have grade 3 toxicity. No grade 4 or 5 toxicity was observed in any patient, and no treatment-related death occurred.

After PS matching, there was no significant difference between regional RT group and local RT group in terms of neutropenia (73.3%vs 53.3%, p = 0.256), grade 3–4 neutropenia (20.0% vs. 13.3%, p = 0.624), grade 3–4 thrombocytopenia (13.3% vs. 6.7%, p = 0.543), anemia (86.7% vs. 66.7%, p = 0.195), grade 3–4 anemia (20.0% vs. 6.7%, p = 0.283), nausea and vomiting (66.7% vs. 60.0%, p = 0.705), grade 3–4 nausea and vomiting (6.7% vs. 6.7%, p = 1.0), radiation enteritis (60.0% vs. 66.7%, p = 0.705), grade 3–4 enteritis (0% vs. 0%, p = 1.0), and grade 3–4 radiation cystitis (0% vs.0%, p = 1.0). However, in terms of thrombocytopenia (60% vs.20%, p = 0.025) and radiation cystitis (60% vs.13.3%, p = 0.008), the regional RT group was greater than the local RT group (Table 5).

**Table 5:** Adverse events between regional RT group and local RT group after matching

Adverse Effect	Patients (%)		
	Regional RT (15)	Local RT (15)	P value
**Neutropenia**	11(73.3)	8(53.3)	0.256
G3-4	3(20.0)	2(13.3)	0.624
**Thrombocytopenia**	9(60.0)	3(20.0)	0.025
G3-4	2(13.3)	1(6.7)	0.543
**Anemia**	13(86.7)	10(66.7%)	0.195
G3-4	3(20.0)	1(6.7%)	0.283
**Radiation enteritis**	9(60.0)	10(66.7%)	0.705
G3-4	3(20.0)	1(6.7%)	0.283
**Radiation cystitis**	9(60.0)	2(13.3)	0.008
G3-4	0(0)	0(0)	1.0
**nausea and vomiting**	10(66.7%)	9(60.0)	0.705
G3-4	1(6.7%)	1(6.7%)	1.0
**fatigue**	14(93.3)	11(73.3)	0.142
G3-4	0(0)	0(0)	1.0

Among 72 patients, 25 patients progressed, while 17 of them died. There were 19 patients with distant metastasis such as liver, lung, extra-regional lymph node, and bone. Four patients had recurrence in situ and two patients had retroperitoneal lymph node metastasis.

## Discussion

At present, there is no standard treatment for patients with locoregional recurrent cervical cancer. Thus, CCRT should be considered with the selective use of brachytherapy [38, 39]. A retrospective study of 50 patients with isolated para-aortic lymph node recurrence of cervical cancer after radical surgery compared the clinical outcomes of different salvage treatment modes, including RT, CCRT, surgery, chemotherapy, and best supportive care. The results showed that CCRT was an effective salvage treatment for isolated para-aortic lymph node recurrence; the 3-year OS and PFS rates associated with CCRT were 85.7% and 71.4%, respectively [11]. Another retrospective study of 22 cervical cancer patients with lymph node recurrence who previously underwent radical operation showed that salvage RT with concurrent chemotherapy was a good choice for patients; the 5-year PFS and OS rates for all 22 patients were 32.7% and 30.7%, respectively [40]. However, these studies did not explore the prognosis following IMRT for patients with local recurrence. A retrospective study comparing IMRT with conventional RT for patients with recurrent cervical cancer found that IMRT included higher irradiation doses for tumors (61.8 Gy vs. 50.3 Gy), had fewer side effects, and resulted in better prognosis (5-year OS: 35.4% vs. 21.4%; 5-year PFS: 26.1% vs. 15.1%) than conventional RT [12]. In our study, all patients received IMRT for external irradiation. The 5-year OS and PFS rates were 65.9% and 57.6%, respectively. results showed that IMRT-based salvage treatment resulted in better prognosis and the 5-year OS and PFS rates were 65.9% and 57.6%, respectively. It was suggested that in the era of IMRT, patients with locoregional recurrence had a better prognosis compared with the era of conventional radiotherapy.

The location of recurrence and metastasis is an important factor influencing prognosis. One study showed that the prognosis of para-aortic lymph node metastasis was better than that of supraclavicular lymph node metastasis and that the prognosis of lymph node metastasis was better than that of hematogenous metastasis [23]. In our study, the pelvic cavity was the most common recurrence site, followed by the vaginal stump and extra-pelvic area. Furthermore, the prognosis of patients with vaginal stump recurrence was better than that of patients with pelvic and extra-pelvic area recurrence. Patients with vaginal stump recurrence generally received a combination of external-beam RT and brachytherapy, which could achieve higher doses and better safety. Therefore, the prognosis of patients with vaginal stump recurrence is better.

Whether the time to recurrence is related to prognosis is questionable. Jeon et al. reported that a time to recurrence of > 18 months was associated with better OS and PFS [40]. Singh et al. reported that the time to recurrence > 24 months after the initial therapy was a good prognostic factor [37]. Another study showed that time to recurrence of > 10 months was not significantly associated with OS [24]. Kubota et al. reported that a median time to recurrence of > 10 months, as well as other cutoff values including 10, 18, and 24 months, was not significantly associated with OS, LC, or PFS [11]. This study showed that the cutoff time to recurrence, whether 21 or 24 months, did not influence prognosis. Further research is needed to verify the relationship between recurrence time and prognosis.

There are many types of salvage RT for locoregional recurrent cervical cancer, and these can be summarized as follows: regional RT and local RT [11, 23, 24]. It is unclear as to which mode is better. Sato et al. compared the effects of the two RT modes on the prognosis of patients with oligo-recurrence; 4 patients received gross tumor RT, whereas 17 patients received RT including the regional lymph node area. No significant difference in outcomes was found [26]. However, the number of cases in this study was very small. This study suggested that regional RT was associated with better prognosis than local RT before and after matching. However, there was no significant difference between them in terms of PFS rate after matching, which may be related to the small sample. Moreover the results may not necessarily be accurate, since the matching analysis of 15 patients was not appropriate. We need to expand the sample size for further analysis. However, at least this matching analysis can provide some point of reference.

Both SIRI and PLR are biomarkers of systemic inflammatory response, which can reflect the tumor microenvironment. Many studies have shown that SIRI, a new systemic inflammatory response biomarker, is a better prognostic factor than other biomarkers. A high SIRI was associated with poor prognosis of patients with various malignancies, and the cut-off value of SIRI used for predicting prognosis was not fixed, and the cut-off value of SIRI varies for different studies [30, 31, 41–43]. In addition, SIRI can change dynamically with changes in tumor burden and immune response status in cervical cancer patients, and patients with a decrease in SIRI of > 75% had better prognosis (p < 0.001). SIRI was also a potential marker for therapeutic response monitoring in patients with curable cervical cancer [29].

A study has found that an elevated SIRI was related to persistent HPV disease, which might be one of the reasons why SIRI affected the prognosis of cervical cancer [44]. Another study constructed a prognostic model including SIRI, and through enrichment analysis, it was found that tumor neutrophil albumin related genes may be involved in the following pathways: cell cycle, homologous recombination, P53 signaling pathway, pyrimidine metabolism, etc. Therefore, Siri may affect the development of tumors by influencing the these signaling pathways through neutrophil related genes [45]. It was known that SIRI was based on neutrophil count. Studies have shown that tumor related neutrophils participate in the tumor microenvironment through cytokines and chemokines, and stimulate immune suppression, tumor growth, angiogenesis, and metastasis through DNA instability or through cytokine and chemokine activity [46]. There is still controversy about the mechanism by which SIRI affects tumor prognosis. PLR was associated with prognosis but was not an independent prognostic factor in many types of cancers [29, 31, 43]. Furthermore, this study showed that SIRI was an independent prognostic factor for patients with locoregional recurrent cervical cancer but not PLR, which was consistent with previous results.

Our study has some limitations. First, this study was a retrospective study. Second, this study involved a relatively small number of patients. Future studies should consider including a larger sample size or employing a randomized clinical study design to validate these preliminary results.

In conclusion, patients with locoregional recurrent cervical cancer who received IMRT-based salvage CCRT had good prognosis. Vaginal stump recurrence, regional RT, only 1 metastatic lesion and a low SIRI predict better survival. Recurrence site and SIRI were independent prognostic factors. Regional RT may be a better option for patients with locoregional recurrent.

### Electronic supplementary material

Below is the link to the electronic supplementary material.

Supplementary Material 1

Supplementary Material 2

## Data Availability

The authors agree to share anonymized data upon reasonable request by researchers. Someone wants to request the data from this study, please contact the corresponding author.
