# Supplementary material for: Salvage radiotherapy strategy and its prognostic significance for patients with locoregional recurrent cervical cancer after radical hysterectomy: a multicenter retrospective 10-year analysis
Source: BMC Cancer. 2023 Sep 26;23:905. doi: 10.1186/s12885-023-11406-z (PMC10521426; doi:10.1186/s12885-023-11406-z)
Supplement: Supplementary file 1 — Supplementary Material 1 [file 12885_2023_11406_MOESM1_ESM.docx]

Supplement file 2 Multivariate analysis of clinical outcomes

| Factor | Multivariate analysis | | |
| --- | --- | --- | --- |
|  | HR | 95% CI | P value |
| **PFS** |  |  |  |
| Radiotherapy Mode | 0.848 | 0.355-2.028 | 0.711 |
| Recurrence Sites | 0.356 | 0.075-1.698 | 0.195 |
| SIRI | 0.347 | 0.134-0.898 | 0.029 |
| No. of recurrence | 0.812 | 0.272-2.426 | 0.709 |
| **OS** |  |  |  |
| Radiotherapy Mode | 0.485 | 0.178-1.320 | 0.157 |
| Recurrence Sites | 0.098 | 0.011-0.922 | 0.042 |
| SIRI | 0.376 | 0.121-1.170 | 0.091 |
| No. of recurrence | 1.490 | 0.461-4.815 | 0.506 |
