# Supplementary material for: Salvage radiotherapy strategy and its prognostic significance for patients with locoregional recurrent cervical cancer after radical hysterectomy: a multicenter retrospective 10-year analysis
Source: BMC Cancer. 2023 Sep 26;23:905. doi: 10.1186/s12885-023-11406-z (PMC10521426; doi:10.1186/s12885-023-11406-z)
Supplement: Supplementary file 2 — Supplementary Material 2 [file 12885_2023_11406_MOESM2_ESM.pdf]

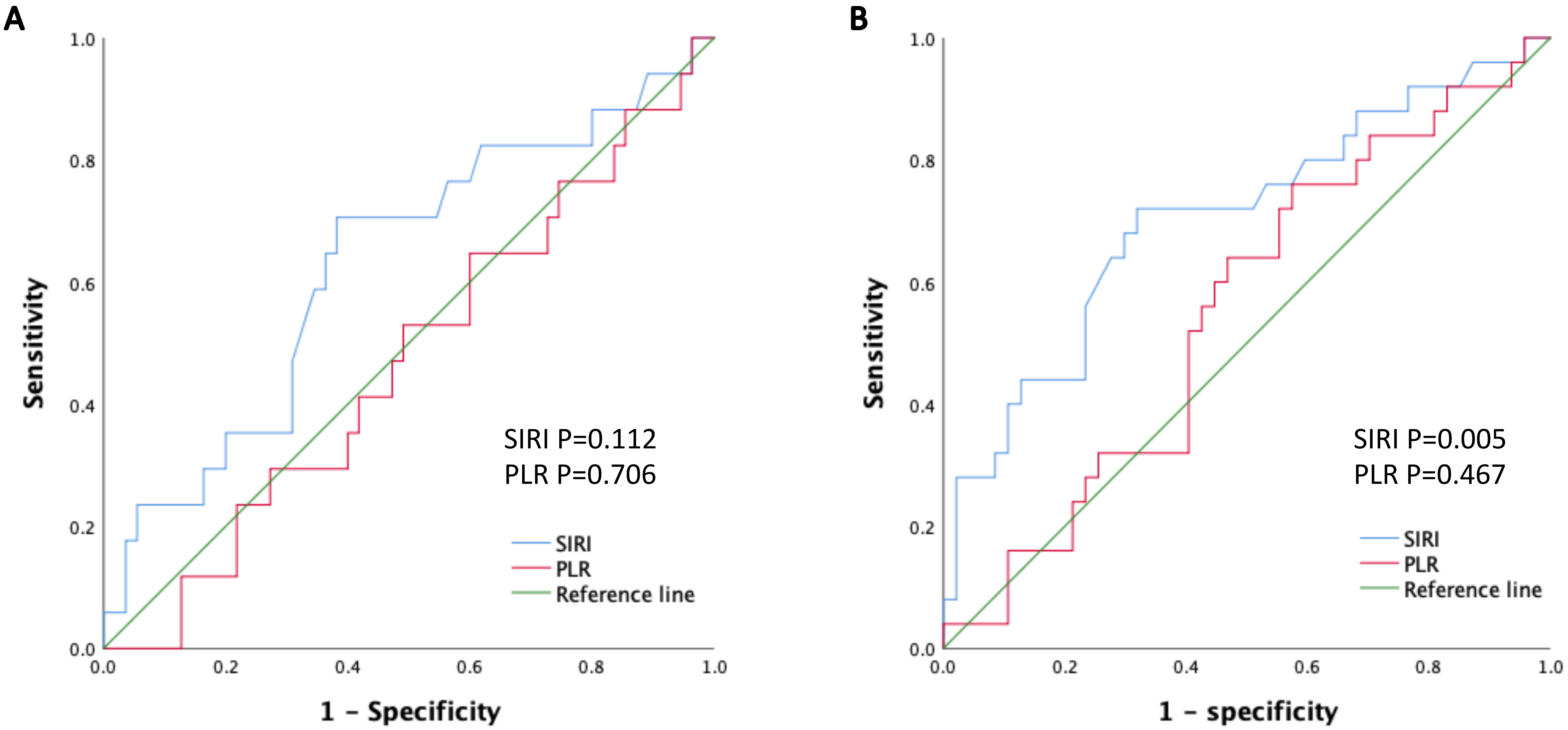

Supplementary file 1: The receiver operating characteristic (ROC) curves of SIRI and PLR based on (a) Overall survival and (b) progression-free survival.
